# Alkaloids Modulate the Functioning of Ion Channels Produced by Antimicrobial Agents via an Influence on the Lipid Host

**DOI:** 10.3389/fcell.2020.00537

**Published:** 2020-06-30

**Authors:** Svetlana S. Efimova, Anastasiia A. Zakharova, Olga S. Ostroumova

**Affiliations:** Laboratory of Membrane and Ion Channel Modeling, Institute of Cytology, Russian Academy of Sciences, St. Petersburg, Russia

**Keywords:** alkaloids, lipid bilayers, ion channels, lipid melting, membrane dipole potential, curvature stress

## Abstract

It is widely recognized that an alteration in membrane physical properties induced by the adsorption of various drugs and biologically active compounds might greatly affect the functioning of peptides and proteins embedded in the membrane, in particular various ion channels. This study aimed to obtain deep insight into the diversity of the molecular mechanisms of membrane action of one of the most numerous and extremely important class of phytochemicals, the alkaloids. Protoalkaloids (derivatives of β-phenylethylamine, benzylamines, and colchicines), heterocyclic alkaloids (derivatives of purine, quinolysidine, piperidine, pyridine, quinoline, and isoquinoline), and steroid alkaloids were tested. We evaluated the effects of 22 compounds on lipid packing by investigating the thermotropic behavior of membrane lipids and the leakage of a fluorescent marker from unilamellar lipid vesicles. The alteration in the transmembrane distribution of the electrical potential was estimated by measuring the alkaloid induced changes in the boundary potential of planar lipid bilayers. We found that benzylamines, the chili pepper active components, capsaicin and dihydrocapsaicin, strongly affect not only the elastic properties of the lipid host, but also its electrostatics by dramatic decrease in membrane dipole potential. We concluded that the increase in the conductance and lifetime of gramicidin A channels induced by benzylamines was related to alteration in membrane dipole potential not to decrease in membrane stiffness. A sharp decrease in the lifetime of single ion pores induced by the antifungal lipopeptide syringomycin E, after addition of benzylamines and black pepper alkaloid piperine, was also mainly due to the reduction in dipole potential. At the same time, we showed that the disordering of membrane lipids in the presence of benzylamines and piperine plays a decisive role in the regulation of the conductance induced by the antifungal polyene macrolide antibiotic nystatin, while the inhibition of steady-state transmembrane current produced by the antimicrobial peptide cecropin A was attributed to both the dipole potential drop and membrane lipid disordering in the presence of pepper alkaloids. These data might lead to a better understanding of the biological activity of alkaloids, especially their action on voltage-gated and mechanosensitive ion channels in cell membranes.

## Introduction

The diversity of the biological effects of phytochemicals, including alkaloids, ensures their huge therapeutic potential: they have been demonstrated to have antimalarial, antimicrobial, antitumor, antidiabetic, antihypertensive, antiarrhythmic, anticholestatic, anesthetic, analgesic, anti-inflammatory, immunomodulating, neuroprotective and many other pharmacologically beneficial properties. Thousands of publications per year devoted to the effects of alkaloids make it practically impossible to overview their pharmacological significance in this paper. For illustration, we cite some selected works from recent years: Clark and Lee (2016), Isah (2016), Kluska et al. (2016), Sultana and Asif (2017), Tsuchiya (2017), Hussain et al. (2018), Shin et al. (2018), Tiwari et al. (2018), Alasvand et al. (2019), Patil et al. (2019), Dembitsky et al. (2020), Li et al. (2020), Rasouli et al. (2020), Shinjyo et al. (2020), Tao et al. (2020), and Tew et al. (2020).

Alkaloids are characterized by significant structural diversity. They are usually divided into several major groups (Aniszewski, 2007). Here, we tested both heterocyclic alkaloids (derivatives of purine, quinolizidine, pyridine, tropane, quinoline, isoquinoline, piperidine, and indole alkaloids), and protoalkaloids with the nitrogen in the side chain (colchicine, benzylamines, and β-phenylethylamine derivatives). Pseudoalkaloids of the steroid type were also tested.

Accumulated literature data suggest that the mechanisms of the influence of alkaloids are very diverse; however, in most cases, the cell membrane might be the primary target of action. The amphiphilic nature of alkaloids might contribute to their incorporation into cell membranes, alterations in the physicochemical properties of the membrane lipid bilayer, and, consequently, in the activity of various receptors, ion channels, and enzymes. This hypothesis can be verified using artificial lipid membranes. In particular, it is widely recognized that some alkaloids might affect the elastic characteristics of the lipid environment. For example, using NMR spectroscopy, it was established that quinine causes a significant drop in the melting temperature of dipalmitoylphosphatidylcholine (DPPC) due to its deep intercalation into the bilayer and significant disordering of the acyl chains of membrane lipids (Zidovetzki et al., 1989). Small-angle X-ray diffraction data and fluorescence polarization measurements showed the DPPC transition from a gel to the interdigitated phase under the action of atropine (Hao et al., 1998). Using numerous methods including differential scanning microcalorimetry, X-ray diffraction, fluorescence probe spectroscopy and NMR, it was found that capsaicin significantly affects the phase behavior of phosphocholines and phosphoethanolamines (Aranda et al., 1995; Torrecillas et al., 2015). In detail, capsaicin significantly reduces the temperature and the cooperatively of dimyristoylphosphocholine (DMPC) and DPPC transition from the ripple to the fluid phase (Aranda et al., 1995; Swain and Mishra, 2015). The observed deconvolution of the main peaks indicates the existence of several mixed alkaloid-lipid phases at high alkaloid concentrations. Moreover, capsaicin provokes the formation non-lamellar inverted hexagonal phases by phosphoethanolamines, probably due to the induction of negative spontaneous curvature of lipid monolayers (Aranda et al., 1995; Ingolfsson and Andersen, 2010). Using molecular modeling and X-ray diffraction, it was established that caffeine interacts with model membranes composed of phosphocholines and phosphoglycerols (Paloncýová et al., 2013; Khondker et al., 2017). The authors demonstrated that the xanthine molecules are located in the region between lipid “heads” and “tails.” The interaction of the alkaloid with the water molecules associated with the neighboring lipids leads to a local increase in bilayer hydration, an increase in the thickness of the membrane and a decrease in its fluidity (Khondker et al., 2017). Authors suggest that the non-specific interaction of caffeine with the lipid bilayer should affect the membrane activity of anesthetics. This assumption is consistent with the data of calorimetry and molecular dynamics showing that caffeine significantly compensates for the disordering effect of tetracaine (Sierra-Valdez et al., 2013). Differential scanning calorimetry data showed that the alkaloid solasodine affects the cooperativity of the phase transition of DMPC, dimyristoylphosphatidylethanolamine and dimyristoylphosphatidylserine in a concentration-dependent manner (Manrique-Moreno et al., 2014). Thus, it has been determined that alkaloid disorders the membrane lipid bilayer.

**FIGURE 1:**
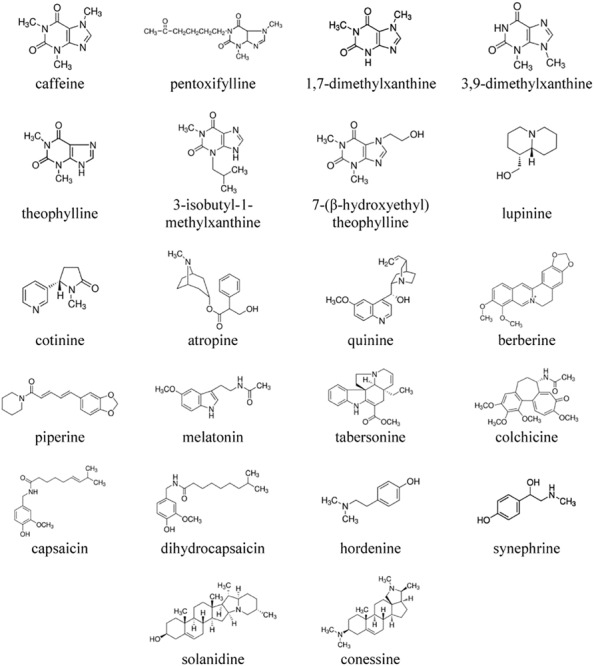
The chemical structures of the tested alkaloids.

It was previously shown that, in addition to the pronounced effects on the elastic properties of membranes, another phytochemicals, i.e., flavonoids, can significantly affect the distribution of electric potentials in the bilayer, and consequently, the ion channels (Luchian and Mereuta, 2006; Asandei et al., 2008; Mereuta et al., 2008, 2011; Efimova and Ostroumova, 2012; Ostroumova et al., 2013, 2015; Efimova et al., 2014a, 2018c). To better understand the molecular mechanisms of alkaloid action, here we have evaluated the quantitative changes in the various physical characteristics of membrane, boundary potential and lipid packing, upon the adsorption of different alkaloids. The effects of 22 compounds (Figure 1) were carefully documented. We also examined the role of the observed changes in the elastic and electric properties of the lipid microenvironment in the function and regulation of ion channels induced by antimicrobial agents.

## Results and Discussion

### Membrane Electrostatics

#### Membrane Boundary Potential

**FIGURE 2:**
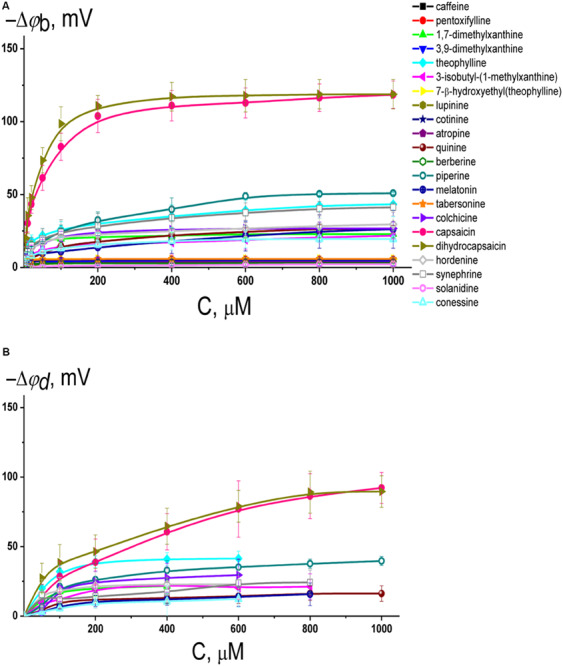
**(A)** Dependence of the decrease in the boundary potential of the membrane (–**Δ**φ_b_) on the concentration (*C*) of different alkaloids in the membrane bathing solution. The membranes were made from POPC, bathed in 0.1 M KCl, pH 7.4, and modified by nonactin. *V* = 50 mV. The relation between the color symbol and the compound is given on the figure. **(B)** Dependence of the reduction in the dipole potential of the POPC membranes (–**Δ**φ_d_) on the concentration in the liposome bathing solution (*C*) of different alkaloids having the distinguishable boundary potential modifying ability (>10 mV). The membranes were modified with the dipole-sensitive fluorescence probe di-8-ANEPPS. The relation between the color symbol and the compound is the same as on panel **(A)**.

Figure 2A illustrates the dependence of the magnitude of reduction in the boundary potential of bilayers composed of pure POPC and bathed in 0.1 M KCl (5 mM HEPES-KOH, pH 7.4) on the concentration of different alkaloids. According to the effects, alkaloids should be divided into three groups: those with pronounced modifying properties (dihydrocapsaicin, and capsaicin), with moderate or weak effects (piperine, theophylline, synephrine, hordenine, colchicine, quinine, melatonin, 1,7-dimethylxanthine, 3-isobutyl-1-methylxanthine, and conessine), and those that have practically no ability to influence the membrane boundary potential (cotinine, tabersonine, 7-(β-hydroxyethyl)theophylline, atropine, 3,9-dimethylxanthine, pentoxifylline, lupinine, berberine, caffeine, and solanidine). One may notice that the dependences are close to linear at low alkaloid concentrations and tend toward saturation at high concentrations. Earlier, we showed that the adsorption of different membrane modifiers, including plant flavonoids, thyroid hormones, and xanthene dyes, onto phospholipid bilayers is adequately described by a Langmuir adsorption isotherm with characteristic parameters: the maximum changes in the membrane boundary potential at an infinitely high concentration of membrane modifier [−Δφ_b_(max)] and the desorption/dissociation constant, which represents a meaningful number for the affinity of membrane modifier to the lipid (*K*) (Efimova and Ostroumova, 2012; Efimova et al., 2014b, 2018c). This approach has been successfully applied to the data presented in Figure 2A. Table 1 shows the values of −Δφ_b_(max) and *K*. Obtained *K*-values are in the range from 10 to 80 μM indicating that alkaloids are able to reduce the boundary potential of lipid bilayers at an order of magnitude higher concentrations than plant flavonoids (the constants *K* of alkaloids are an order of magnitude higher compared to those of flavonoids) (Efimova and Ostroumova, 2012, 2015; Ostroumova et al., 2013; Efimova et al., 2014b, 2018c). This discrepancy cannot be explained in terms of differences in the lipophilicity of plant metabolites expressed in the logarithm of the octanol/water partition coefficients (Log*P*_o/w_); for example, the most effective agents among flavonoids and alkaloids, phloretin and capsaicin, are characterized by close logarithms of the octanol/water distribution coefficients at pH 7.4 (Log*D*_o/w_) predicted by ChemAxon calculation (3.79 and 3.75, respectively). The dissociation constant *K* calculated from the φ_b_-changes describes only the electrical aspects of adsorption of the molecules to lipid membranes and does not take into account the adsorption, which does not lead to a change in the potential jump, in particular, at a high surface density, as well as preferable localization of modifiers in the lipid bilayer or their transmembrane permeation (Cseh et al., 2000).

**TABLE 1:** The parameters characterized the effects of alkaloids on the physical properties of lipid bilayers: Δ*φ_b_*(max) – the maximum changes in the boundary potential of POPC-membranes, *K* – the desorption constant of alkaloid, Δφ_*d*_(max) – the maximum changes in the dipole potential of POPC-membranes; Δ*T*_*p*_ – the changes in the DPPC pre-transition temperature, Δ*T*_m_ – the changes in the main transition temperature; Δ*T*_1/2_ – the changes in the half-width of the main peak at a lipid:alkaloid molar ratio of 10:1; *RF*_max_ – maximal leakage of calcein from unilamellar POPC vesicles.

**Alkaloid**	**POPC**			**DPPC**			**POPC**
	−Δ**φ_b_(max), mV**	***K*, μM**	−Δ**φ_*d*_(max), mV**	**Δ*T*_*p*_, °C**	**−**Δ***T*_m_, °C**	**Δ*T*_1/2_, °C**	***RF_max_, %***
*Caffeine*	2 ± 2	*na*^$^	*na*	0	0	0	3 ± 1
*Pentoxifylline*	4 ± 2	*na*	*na*	0	0	0	2 ± 1
**1,7-Dimethylxanthine**	23 ± 5	11 ± 7	21 ± 6	0	0	0	5 ± 1
*3,9-Dimethylxanthine*	4 ± 3	*na*	*na*	0	0	0	4 ± 1
*Theophylline*	41 ± 16	42 ± 16	40 ± 5	–*	0.2 ± 0.1	0.1 ± 0.1	6 ± 1
*3-Isobutyl-1-methylxanthine*	22 ± 3	33 ± 13	20 ± 9	–*	0.9 ± 0.1	0.1 ± 0.1	7 ± 2
**7-(β-hydroxyethyl) theophylline**	6 ± 2	*na*	*na*	0	0	0	4 ± 1
*Lupinine*	3 ± 3	*na*	*na*	0	0.5 ± 0.1	0.1 ± 0.1	5 ± 2
*Cotinine*	6 ± 2	*na*	*na*	0	0	0	3 ± 1
*Atropine*	4 ± 4	*na*	*na*	0	0	0	3 ± 1
*Quinine*	26 ± 9	80 ± 10	16 ± 6	–*	0.6 ± 0.1	0.7 ± 0.1	12 ± 1
*Berberine*	3 ± 2	*na*	*na*	–*	0.3 ± 0.3	0.1 ± 0.1	12 ± 3
*Piperine*	51 ± 8	79 ± 23	40 ± 13	–*	2.9 ± 0.3	0.9 ± 0.2	31 ± 9
*Melatonin*	26 ± 9	37 ± 9	15 ± 8	–*	0.7 ± 0.1	0.1 ± 0.1	2 ± 1
*Tabersonine*	6 ± 2	*na*	*na*	–*	0.2 ± 0.1	2.4 ± 0.4	81 ± 5
*Colchicine*	27 ± 5	19 ± 3	29 ± 11	2.5 ± 0.8	0.8 ± 0.3	0.9 ± 0.1	4 ± 2
*Capsaicin*	118 ± 11	32 ± 7	92 ± 11	–*	4.8 ± 0.6	3.6 ± 0.9	57 ± 5
**Dihydrocapsaicin**	119 ± 12	26 ± 6	92 ± 15	–*	4.1 ± 0.2	2.2 ± 0.3	66 ± 4
*Hordenine*	29 ± 8	19 ± 2	23 ± 11	3.0 ± 0.6	1.0 ± 0.2	0.1 ± 0.1	6 ± 1
**Synephrine**	41 ± 12	44 ± 6	24 ± 9	3.2 ± 0.2	0.8 ± 0.1	0.1 ± 0.1	6 ± 1
**Solanidine**	2 ± 2	*na*	*na*	–*	0.9 ± 0.2	1.1 ± 0.1	4 ± 2
*Conessine*	19 ± 6	29 ± 14	12 ± 7	–*	1.8 ± 0.4	0.7 ± 0.1	30 ± 2

^$^*na*, not applicable. *The pre-transition was suppressed.

#### Membrane Dipole Potential

It should be noted that the boundary potential of the membrane (which can be estimated by measuring K^+^-nonactin induced membrane conductance of planar lipid bilayers) consists of two components: the surface potential, φ_s_, which is generated by the charged head groups of phospholipids and the adsorbed ions and charged molecules at the interface, and the dipole potential, φ_*d*_, which is related to the chemical structure of the interface, orientation, and hydration of the polar lipid headgroups (Gawrisch et al., 1992; Wang, 2012; Ermakov and Nesterenko, 2017). The measurements of the dipole potential using a dipole-potential-sensitive lipid fluorescence probe, di-8-ANEPPS (Gross et al., 1994), may provide additional information regarding which component of the membrane boundary potential is responsible for the regulation of ion transport through membranes after the adsorption of alkaloids that are characterized by the ability to modify the membrane boundary potential. Figure 2B presents the dependences of the diminution in dipole potential of POPC bilayers upon the adsorption of tested alkaloids. The maximal decrease in φ_*d*_ upon addition of dihydrocapsaicin, capsaicin, piperine, theophylline, synephrine, quinine, hordenine, melatonin, colchicine, 3-isobutyl-1-methylxanthine, 1,7-dimethylxanthine, and conessine are presented in Table 1. Comparing Δφ_b_(max) and Δφ_*d*_(max) absolute values one can conclude that dipole potential makes the main contribution to the potential drop at the interface after the adsorption of alkaloids, despite the fact that some of tested alkaloids have a nonzero charge at pH 7.4 basing on their pKa-values (Supplementary Table 1S).

### The Relation of the Dipole-Modifying Ability to the Lipophilicity and Polarity of Alkaloid Molecules

The octanol/water partition coefficient (or the distribution coefficient referring to the ionized species) and the dipole moment are generally used as a measure of lipophilicity and polarity of organic molecules. Supplementary Table 1S presents the logarithm of octanol/water distribution coefficients of tested alkaloids at pH 7.4, Log*D*_o/w_, predicted by ChemAxon, and the logarithm of their octanol/water partition coefficients at various conditions, Log*P*_o/w_, found in the literature. The correlation coefficients between Log*D*_o/w_ and −Δφ_b_(max) or −Δφ_*d*_(max) values are 0.75 and 0.78, respectively. Relationships between Log*P*_o/w_ and −Δφ_b_(max) or −Δφ_*d*_(max) values are characterized by slightly lower coefficients. Thus, a significant correlation is observed between the dipole-modifying ability and lipophilicity of alkaloids.

In addition to the affinity to the lipid phase, which determines the alkaloid concentration in the membrane, the dipole-modifying properties should depend on the polarity of adsorbed molecules. Supplementary Table 1S also shows the dipole moments of tested alkaloids calculated by HyperChem and their comparison with the available literature data. No correlation was found between the dipole moments of alkaloids, μ, and −Δφ_b_(max) or −Δφ_*d*_(max) values. The main reason for this discrepancy should be the prevailing role of the molecule and dipole moment vector orientation in the bilayer, and the depth of immersion of the alkaloid molecules into membrane. To support the assumption, the molecular dynamic simulations evaluated that the disordering and dipole-modifying effects of local anesthetics on the lipid bilayers depended on the preferential molecular location and orientation relative to membrane normal (Hogberg et al., 2007; Hogberg and Lyubartsev, 2008; Mojumdar and Lyubartsev, 2010; Zapata-Morin et al., 2014). Furthermore, the increase in dipole potential of the membrane induced by various styryl dyes of the RH series, RH 160, RH 237, RH 421, was associated with the depth of chromophore immersion into the bilayer (Malkov and Sokolov, 1996; Passechnik and Sokolov, 2002). Applying high-resolution magic angle spinning NMR spectroscopy Scheidt et al. (2004) investigated the membrane localization/orientation of flavonoid molecules which can define the antioxidant activity of plant polyphenolic compounds.

Thus, the pronounced boundary and dipole potential modifying ability of benzylamines, capsaicin and dihydrocapsaicin (about 100 mV, Table 1) might be presumably associated with the high lipophilicity and polarity of these molecules (Supplementary Table 1S), their orientation along the bilayer normal, and considerable immersion depth of these molecules into the bilayer (the vanillyl groups of capsaicin and dihydrocapsaicin interact with the carbonyls of lipid molecules) (Hanson et al., 2015).

A noticeable drop in the membrane boundary/dipole potential (20–50 mV, Table 1) in the presence of piperine, quinine, melatonin and colchicine should be also related to their marked lipophilicity and polarity (Supplementary Table 1S).

Comparing the dipole modifying properties of the xanthine derivatives, one may notice that caffeine (trimethylxanthine), pentoxifylline (3,7-dimethyl-1-(5-oxohexyl)xanthine), 7-(β-hydroxyethyl)theophylline, and 3,9-dimethylxanthine did not affect the membrane boundary/dipole potential (Table 1). Herewith, 1,7-dimethylxanthine, theophylline (1,3-dimethylxanthine), and 3-isobutyl-1-methylxanthine decreased φ_b_ and φ_*d*_ by 20–40 mV. Taking into account the significant dipole moments and the modest octanol/water partition coefficients of the xanthine derivatives (Supplementary Table 1S), one can conclude that the membrane-modifying effect is probably not related to the number and origin of the groups introduced into the xanthine structure, but rather due to the different orientation of the various derivatives in the bilayer. For example, trimethylxanthine (caffeine) was not characterized by the greater ability to decrease membrane boundary potential compared to dimethylxanthines (1,7-dimethylxanthine, 3,9-dimethylxanthine, and theophylline). Moreover, the introduction of two methyl groups into the different positions of xanthine core of the molecules of 1,7-dimethylxanthine, 3,9-dimethylxanthine, and theophylline led to strictly different dipole-modifying activity of the dimethylxanthines. Replacement of the methyl to more hydrophobic isobutyl group (theophylline vs. 3-isobutyl-1-methylxanthine) did not lead to an increase in the dipole-modifying ability of the derivative, but, on the contrary, caused its reduction. It is likely that the introduction of different groups into the highly polar xanthine molecule changes its orientation in the membrane, and, consequently, the projection of its dipole moment onto the normal to the surface of the lipid bilayer, which contributes to the changes in the boundary and dipole potential.

Probably, the orientation of dipole moment vector of steroid molecule in the membrane is also the key to understanding the different dipole-modifying ability of solanidine and conessine (Table 1) at almost the same polarity of the molecules and higher lipophilicity of inactive solanidine compared to more effective conessine (Supplementary Table 1S).

Similar to xanthine and steroid derivatives, a possible explanation for the distinguishable ability of 2-phenylethylamine derivatives, synephrine and hordenine, to affect the electric potential drop at the membrane-water interface (20–40 mV, Table 1) at the relatively low partition coefficients (Supplementary Table 1S) is not directly related to the perceptible magnitude of their dipole moments (ineffective cotinine and atropine are characterized by the higher μ-values than 2-phenylethylamine derivatives), but rather refers to the vector orientation in the bilayer.

The significant partition coefficient of tabersonine (about 1, Supplementary Table 1S) indicates that the absence of its influence on the membrane boundary/dipole potential (Table 1) is associated with the orientation of its dipole moments parallel to the membrane plane. Nevertheless, the relatively high lipophilicity of tabersonine suggests that this alkaloid affects other physical properties of the membranes, in particular, lipid packing.

### Lipid Packing

#### Lipid Melting

**FIGURE 3:**
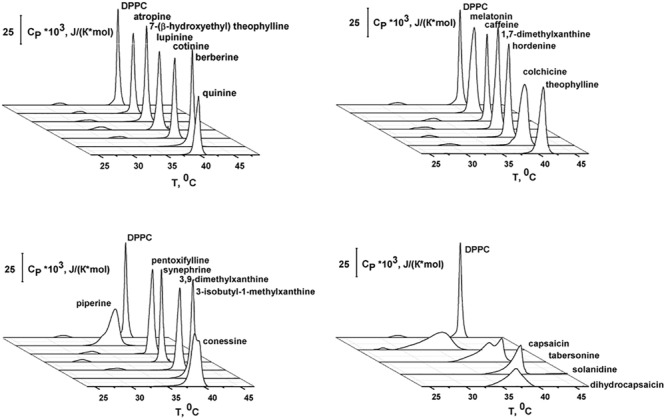
Heating DSC thermograms of DPPC liposomes in the absence (*control*) and presence of different alkaloids at the lipid:agent molar ratio of 10:1.

Differential scanning calorimetry (DSC) heating endotherms illustrating the effects of the tested alkaloids on the thermotropic phase behavior of unilamellar DPPC-vesicles are presented in Figure 3. In the absence of any additives, the endotherm of DPPC exhibited two distinct events: a less energetic pretransition near 33.9°C and a more energetic main or chain-melting transition near 41.3°C. The first one is related to the conversion from the gel (*L*_β_′) to the ripple (*P*_β_′) phase, while the second one arises from the conversion from *P*_β_′ to the liquid crystalline (*L*_α_) phase. Each event might be characterized by the maximum temperature (*T*_*p*_ and *T*_m_, respectively) and the half-width of the peak (*T*_1/2_). The *T*_1/2_ of the second peak is related to the inverse cooperativity of the main transition (a narrow peek indicates complete cooperativity) and it is equal to 0.4°C for DPPC alone. The changes in the temperature of the pretransition (Δ*T*_*p*_) and main transition (Δ*T*_m_) of untreated DPPC vesicles in the absence and in the presence of the tested agents and the half-widths of the main peaks (Δ*T*_1/2_) are presented in Table 1.

From Figure 3 and Table 1, one may notice that the addition of alkaloids differentially affected the pretransition. Caffeine, pentoxifylline 1,7-dimethylxanthine, 3,9-dimethylxanthine, 7-(β-hydroxyethyl)theophylline, lupinine, cotinine, and atropine had practically no influence on *L*_β_′–*P*_β_′ conversion at a lipid/peptide ratio of 10:1. The introduction of colchicine, hordenine, and synephrine significantly shifted the pretransition peak up to lower temperatures, by 2.5–3.2°C. In the presence of theophylline, 3-isobutyl-1-methylxanthine, quinine, berberine, piperine, melatonin, tabersonine, capsaicin, dihydrocapsaicin, solanidine, and conessine, the pretransition was abolished entirely in DPPC vesicles having a lipid/peptide ratio of 10:1. Severcan et al. (2005) also showed a disappearance of pretransition upon the addition of melatonin.

Caffeine, pentoxifylline, 1,7-dimethylxanthine, 3,9-dimethylxanthine, 7-(β-hydroxyethyl)theophylline, cotinine, and atropine did not affect the DPPC main transition at a lipid/peptide ratio of 10:1. These data are in agreement with the findings of Flaten et al. (2007) and Kafaheva et al. (1990) at a lipid/alkaloid ratio of 1.2:1; DSC experiments were performed with caffeine and atropine, respectively. The effects of theophylline, lupinine, and berberine on the *P*_β_′–*L*_α_ DPPC transition were weak (Δ*T*_m_ and Δ*T*_1/2_ were less than 0.5°C). 3-isobutyl-1-methylxanthine, melatonin, hordenine, and synephrine markedly reduced *T*_m_ (on 0.7–1.0°C) and practically did not change *T*_1/2_ (the changes did not exceed 0.2°C). These data are in good accordance to the results of Severcan et al. (2005) who showed that, the main phase transition shifts to lower temperatures as the melatonin concentration is increased. DSC performed by de Lima et al. (2010) indicated that melatonin also induces a shift to a more fluid state of the hydrophobic region of egg phosphocholine even at the higher lipid/alkaloid ratio (880:1). Using Fourier transform infrared spectroscopy, Lebecque et al. (2018) showed that no *T*_m_ change was observed when hordenine was added to multilamellar vesicles made of DMPC. One can notice that among tested xanthine derivatives, only 3-isobutyl-1-methylxanthine demonstrated a significant effect on *T*_m_ which might be due to its hydrophobic branched hydrocarbon side chain.

The addition of tabersonine did not noticeably change *T*_m_ (the changes did not exceed 0.3°C), but increased *T*_1/2_ by 2.4°C. The presence of quinine, piperine, colchicine, capsaicin, dihydrocapsaicin, solanidine, and conessine led to a significant broadening of the main transition peak and a noticeable shift in *T*_m_ toward lower temperatures (both *T*_m_ and *T*_1/2_ were changed by more than 0.6°C). The ability to decrease *T*_m_ increased in the order quinine ≤ colchicine ≤ solanidine < conessine < piperine < dihydrocapsaicin < capsaicin. The ability to increase *T*_1/2_ increased in the order quinine ≈ conessine ≤ colchicine ≤ piperine ≤ solanidine < dihydrocapsaicin < capsaicin. Both effects (decrease in *T*_m_ and increase in *T*_1/2_) should reflect a reduction in membrane order induced by the intercalation of alkaloid molecules between the acyl chains of DPPC, which disrupts their regular packing. The similar conclusion was made by Torrecillas et al. (2015) using DSC, X-ray diffraction, ^31^P NMR, and ^2^H NMR spectroscopy to show that capsaicin increases the fluidity and disorder of DPPC membrane models. The efficiency of alkaloids to disorder membrane lipids should depend on their concentration in the bilayer. In confirmation, the correlation coefficients between Log*D*_o/w_ or Log*P*_o/w_ (Supplementary Table 1S) and −Δ*T*_m_- and Δ*T*_1/2_-values (Table 1) are in the range of 0.74–0.77, demonstrating the good correlation between the alkaloid ability to alter lipid packing and their lipophilicity.

Figure 3 also showed that the main peak in the presence of quinine, piperine, tabersonine, colchicine, capsaicin, dihydrocapsaicin, solanidine, and conessine composed of at least two components. This indicates the presence of some more phases appearing at lower temperatures richer in alkaloids. Moreover, a pronounced main peak deconvolution in the presence of capsaicin and dihydrocapsaicin is accompanied by a detectable decrease in enthalpy change.

#### Lipid Vesicle Leakage

Membrane disordering and a decrease in the lipid packing density should also lead to an increase in bilayer permeability, including the permeability of different fluorescent markers, in particular calcein. To confirm our DSC findings, we measured calcein leakage from large unilamellar vesicles made of POPC induced by different alkaloids (Supplementary Figure 1S). In the presence of 0.4 mM of caffeine, pentoxifylline, 1,7-dimethylxanthine, 3,9-dimethylxanthine, theophylline, 3-isobutyl-1-methylxanthine, 7-(β-hydroxyethyl)theophylline, lupinine, cotinine, atropine, melatonin, colchicine, hordenine, synephrine, and solanidine, the maximum relative release of the fluorescent marker (*RF*_max_) did not exceed 10%. The efficacy to disengage the fluorescence marker from POPC vesicles increased in the following order: quinine ≈ berberine < piperine ≤ conessine < capsaicin ≤ dihydrocapsaicin < tabersonine. Table 1 presents the mean *RF*_max_-values. Comparing effects of quinine, berberine, piperine, conessine, capsaicin, dihydrocapsaicin, and tabersonine on calcein leakage and DPPC melting one can notice a greater agreement between the efficiency of increasing membrane permeability for a fluorescent marker and the alteration in lipid melting cooperativity than between *RF*_max_-values and the changes in the transition temperature. This observation might indicate that the decrease in a cooperativity of the main transition might be related not only to a total decrease in lipid packing density, but also to a membrane curvature stress produced by alkaloid intercalation. The predominant incorporation of alkaloids into the hydrophilic region of the membrane can disrupt the balance between polar and non-polar membrane regions and increase the permeability of the bilayer to calcein. In addition to dependence on concentration of the alkaloids in the membrane, the last effect should be presumably defined by the depth of their immersion. This is probably the reason for the moderate correlation between Log*D*_o/w_/Log*P*_o/w_ and *RF*_max_ values (the coefficient is about 0.61–0.65).

### Reconstituted Ion Channels

A detailed literature analysis of the effects of the tested alkaloids on ion channels in cell membranes has revealed the possibility of their lipid-mediated action (Lundbaek et al., 2005; Søgaard et al., 2006; Ingólfsson et al., 2014; Schmidt et al., 2018). It was found that a decrease in membrane stiffness in the presence of capsaicin is responsible for modulating the activity of voltage-dependent sodium channels (Lundbaek et al., 2005), GABA receptors (Søgaard et al., 2006), and degenerin/epithelial sodium channels such as ASIC1a, ASIC3, ENaC, and P2X2 (Schmidt et al., 2018). Ingólfsson et al. (2014) showed similar effects for mechano-sensitive channels of large conductance (MscL), the voltage-dependent potassium channel Kv2.1, and neuronal voltage-dependent sodium channels (NaV). Aréchiga-Figueroa et al. (2017) also proposed that the effects of capsaicin on Kv2.1 channel gating are mediated by changes in the physicochemical properties. Moreover, Ingólfsson et al. (2014) suggested that the effects of capsaicin on the cystic fibrosis transmembrane conductance regulator (CFTR), large-conductance calcium activated potassium channels (BK/Maxi-K), L-type calcium channels, and TRP channels might be due to cell membrane perturbation rather than specific protein binding. It should be noted that the mechanisms were mainly thought to be related to lipid bilayer elasticity (an increase in density in the head group region, membrane thinning, and increased bending); the modulation of transmembrane electric potentials were not discussed at all.

#### Gramicidin A and Syringomycin E Channels

To investigate the diversity of the mechanisms of alkaloid action on membrane protein function by altering the electrical characteristics of the lipid microenvironment, we used exogenous antimicrobial peptides as molecular force transducers. Among the alkaloids, we chose pentoxifylline, which did not show any effects on the physicochemical properties of the membranes, as well as piperine and synephrine, which were characterized by moderate modulating efficiency, and the alkaloids with the strongest effects on lipid media, capsaicin and dihydrocapsaicin (Table 1). Two ion channel types formed by gramicidin A (GrA) and syringomycin E (SyrE), known to be sensitive to alterations in the transmembrane distribution of electrical potentials, were tested. In model lipid membranes GrA forms channels with almost ideal cation permeability (Finkelstein and Andersen, 1981; Kelkar and Chattopadhyay, 2007). In contrast, SyrE channels are predominantly anion selective (Feigin et al., 1996; Schagina et al., 1998; Malev et al., 2002). These two probes are quite suitable for assessing the modulation of the electrical properties of the membrane by alkaloids. The decrease in dipole potential (with the hydrocarbon region being positive relative to the aqueous phase) is expected to diminish the electrostatic energy at the center of the pore for cations and increase for anions (Andersen et al., 1976). In fact, single-channel data by Rokitskaya et al. (1997) showed that the plant flavonoid phloretin, known to decrease the dipole potential, led to an increase in the amplitude and lifetime of GrA channels. Similar effects were found by Busath et al. (1998) using glycerylmonoolein bilayers with a less positive interfacial potential in the bilayer interior than diphytanoylphosphatidylcholine membranes. Using different dipole modifiers we showed that the reduction in the membrane dipole potential leads to a decrease in SyrE channel conductance and lifetime (Ostroumova et al., 2007).

**FIGURE 4:**
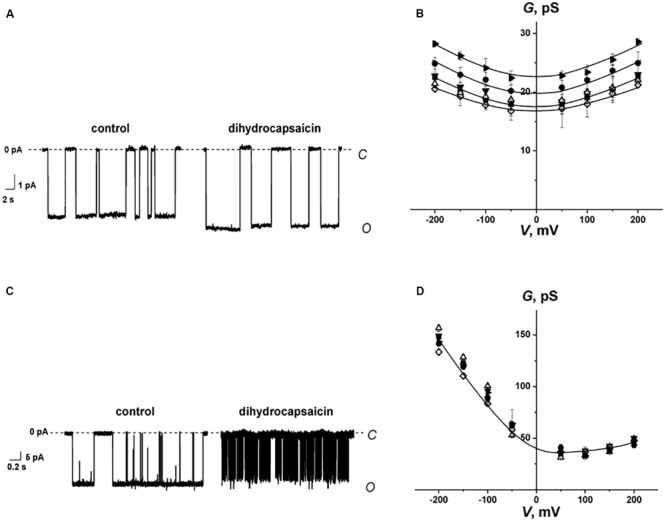
**(A,C)** Current fluctuations corresponding to the openings and closures of single GrA **(A)** and SyrE channels **(C)** in the absence (*control*) and presence of 0.4 mM dihydrocapsaicin. *V* = −200 mV. *C* – closed state of the channel, *O* – open state of the channel. **(B,D)** *G*(*V*) curves of single GrA **(B)** and SyrE channels **(D)** in the absence (■) and presence of 0.4 mM piperine (♢), pentoxifylline (▼), synephrine (Δ), capsaicin (⚫), and dihydrocapsaicin (▶). The membranes were composed of POPC and bathed in 2.0 M KCl (pH 7.4) (A,C) and 1.0 M KCl (pH 7.4) (B,D).

Figures 4A,C show the recordings of current fluctuations corresponding to openings and closures of GrA and SyrE channels in POPC membranes in the absence (control) and in the presence of 0.4 mM dihydrocapsaicin at a transmembrane voltage of −200 mV. One can see that benzylamine led to a slight increase in GrA channel amplitude and an insignificant decrease in SyrE pore amplitude. The shielding effect of the peptide and lipid dielectrics depending on the pore geometry (Jordan, 1983; Malkov and Sokolov, 1996) is the reason for such moderate and weak changes in channel conductance upon the adsorption of dihydrocapsaicin, which reduces the membrane dipole potential on almost 90 mV (Figure 2B and Table 1). About 80% and 95% of dipole potential is shielded in GrA and SyrE channels, respectively (Ostroumova et al., 2008). Table 2 demonstrates the conductance values of GrA and SyrE channels in POPC membranes in the absence and presence of 0.4 mM pentoxifylline, piperine, capsaicin, dihydrocapsaicin, and synephrine at a transmembrane voltage of 200 mV. Figures 4B,D present the total conductance-voltage curves of GrA and SyrE channels before and after addition of tested alkaloids. One can see that alkaloids practically did not affect the *G*-*V* shape of both channel types excluding the interaction of alkaloids with peptides. Table 2 demonstrates that pentoxifylline and synephrine did not affect GrA channel amplitude. The addition of capsaicin and dihydrocapsaicin to the membrane bathing solution led to a 10–20% increase in GrA channel conductance. Thus, we might conclude that changes in bilayer electrostatics under the action of benzylamines underlie the increase in the conductance of GrA pores.

From Figures 4A,C, one can also note an increase in the lifetime of the GrA channels and a sharp decrease in the duration of SyrE pores after the addition of dihydrocapsaicin. The observed effects could also be attributed to the modulation of the membrane dipole potential by the alkaloids. It was shown that, in contrast to the insignificant changes in channel conductance, the lifetime of the channels changed dramatically as a function of dipole potential, with a several-fold increase for GrA channels and a 100-fold reduction for SyrE channels (Rokitskaya et al., 1997; Ostroumova et al., 2007). In full qualitative and quantitative compliance, we measured about a two-fold increase in GrA channel lifetime and a decrease in the magnitude in SyrE pore duration by two orders of under the action of benzylamines (Table 2). The higher sensitivity of the lifetime of SyrE channels to the changes in the dipole potential allowed detecting the effects of piperine and synephrine, which were characterized by less pronounced dipole-modifying properties compared to benzylamines.

#### Cecropin A Channels

**FIGURE 5:**
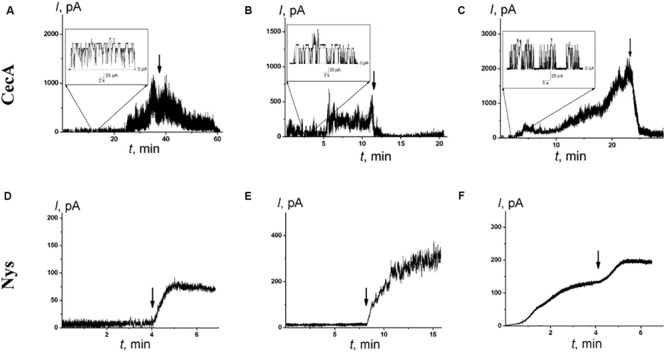
The effects of alkaloids on the steady-state transmembrane current flowing through membranes modified by CecA **(A**–**C)** and Nys **(D–F)**. The moment of the addition of 0.4 mM piperine **(A,D)**, dihydrocapsaicin **(B,E)**, and capsaicin **(C,F)** to the bilayer bathing solution is indicated by an arrow. The lipid bilayers were composed of DOPS/DOPE (50/50 mol%) and bathed in 0.1 M KCl, pH 7.4 (A–C) or POPC/Chol (67/33 mol%) and bathed in 2.0 M KCl, pH 7.4 (D–F). ***V*** = 50 mV. Inserts: Current fluctuations of single CecA channels.

An antibacterial peptide cecropin A (CecA) induces ion channels that are also sensitive to the transmembrane distribution of electric potentials (Christensen et al., 1988; Efimova et al., 2014a). Figures 5A–C demonstrate that pentoxifylline and synephrine did not change the steady-state transmembrane current induced by CecA in DOPS/DOPE bilayers, while piperine, capsaicin, and dihydrocapsaicin inhibited the pore-forming activity of this antimicrobial peptide. Table 2 presents the mean *I*_∞_/*I*_∞_^0^ ratios of the CecA produced steady-state membrane current after and before alkaloid addition. One can see that piperine, capsaicin, and dihydrocapsaicin lead to a 3- to 10-fold decrease in the steady-state transmembrane current induced by CecA, while the mean *I*_∞_/*I*_∞_^0^ upon the addition of pentoxifylline and synephrine was about 1. Recently we have showed that pore-forming ability of CecA depends on the membrane dipole potential; its decrease leads to drop in the effective number of open CecA channels (Efimova et al., 2014a). The effects have been concluded to relate to changes in the energy barrier for the interfacial accumulation of CecA monomers; and the decrease in membrane dipole potential is thought to cumber the immersion of C-terminal peptide helices having partial negative charges (Efimova et al., 2014a). Piperine, capsaicin, and dihydrocapsaicin were characterized by similar effects on the boundary potential of DOPS/DOPE bilayers (60 ± 9, 90 ± 10, and 90 ± 10 mV, respectively) compared to POPC membranes (Table 1). Thus, the observed reduction of *I*_∞_ in the presence of pepper alkaloids was mainly due to the decrease in φ_*d*_. It is believed that CecA forms ion channels by associating peptide monomers into conducting oligomers, which should make the process of pore formation sensitive to membrane stiffness (Christensen et al., 1988; Efimova et al., 2014a, 2018b). Considering the pronounced effect of piperine, capsaicin, and dihydrocapsaicin on the packing density of membrane lipids compared to pentoxifylline and synephrine, the decrease in *I*_∞_ might be also attributed to the destabilization of channel-forming CecA oligomers with a decrease in lipid packing density, which has been observed upon the introduction of some flavonoids, styryl dyes, and local anesthetics (Efimova et al., 2014a, 2018b).

#### Nystatin Pores

To further demonstrate the possibility of the regulation of ion transport via channels due to the influence of alkaloids on lipid bilayer elasticity, we examined the pores induced by the polyene macrolide antibiotic nystatin (Nys). Added from one side of the POPC/Chol membrane, Nys forms single-length channels with a lipid mouth on the opposite side. The presence of a lipid mouth with a positive curvature results in high sensitivity of Nys channels to the membrane curvature stress induced by the adsorption of various agents (Chulkov et al., 2015; Efimova et al., 2018a). Figures 5D–F demonstrate the effects of the addition of piperine, capsaicin, and dihydrocapsaicin into the bathing solution up to 0.4 mM on the Nys-induced steady-state transmembrane current through Chol-enriched membranes (33 mol%). The addition of the tested alkaloids led to a 4- to 10-fold increase in Nys pore-forming activity, while the addition of pentoxifylline and synephrine practically did not affect the Nys-induced steady-state membrane current: the *I_∞_/I_∞_^0^* ratio was about 1 (Table 2). It should be noted that piperine, capsaicin, and dihydrocapsaicin produced significant calcein leakage not only from POPC vesicles (Table 1) but also from POPC/Chol (67/33 mol%) liposomes (*RF*_max_ values are equal to 22 ± 2, 38 ± 2, and 52 ± 16%, respectively). These data might indicate that piperine, capsaicin, and dihydrocapsaicin promote the positive curvature of the lipid monolayer and facilitate pore formation by Nys, while pentoxifylline and synephrine do not. This assumption is in contradiction with previous data (Aranda et al., 1995; Ingolfsson and Andersen, 2010) showing that capsaicin enhances the tendency of phosphoethanolamine to form non-lamellar inverted hexagonal phases, suggesting that its adsorption increases the negative spontaneous curvature of lipid monolayers. However, our data are in agreement with representations of Ingólfsson et al. (2014), who considered an increase in density in the head group region, as well as membrane thinning and increased bending due to the introduction of capsaicin into the membrane.

**TABLE 2:** Regulation of ion channel functioning by different alkaloids: *G*_SC_ – the conductance of single SyrE- or GrA-channels in the absence and presence of 0.4 mM alkaloid at *V* = 200 mV; τ – the dwell time of SyrE or GrA channels; *I_∞_/I_∞_^0^* – the ratio of the transmembrane current induced by the CecA or Nys in the presence and in the absence of alkaloids at *V* = 50 mV.

**Alkaloid**	**POPC**		**POPC**		**DOPE/DOPS**	**POPC/Chol**
	**SyrE**		**GrA**		**CecA**	**Nys**
	***G_SC_, pS***	***τ, ms***	***G_SC_, pS***	***τ, s***	***I_∞_/I_∞_^0^***	***I_∞_/I_∞_^0^***
*Control*	49 ± 1	114 ± 4	22.5 ± 0.6	3.0 ± 1.0	–	–
*Pentoxifylline*	50 ± 2	140 ± 9	23.0 ± 0.8	3.5 ± 0.9	0.9 ± 0.1	1.0 ± 0.1
*Piperine*	47 ± 2	5 ± 1	22.0 ± 1.1	2.7 ± 1.1	0.3 ± 0.1	4.5 ± 0.9
*Capsaicin*	45 ± 3	1 ± 1	25.0 ± 1.0	5.9 ± 2.0	0.1 ± 0.1	4.1 ± 1.0
*Dihydrocapsaicin*	46 ± 2	1 ± 1	26.6 ± 0.8	4.8 ± 1.0	0.2 ± 0.1	9.8 ± 2.3
*Synephrine*	49 ± 2	67 ± 2	22.0 ± 0.8	2.8 ± 1.1	1.1 ± 0.4	1.2 ± 0.1

*The lipid composition of bilayers is presented in this table. The membranes modified by SyrE, GrA, CecA, and Nys were bathed in 2.0, 1.0, 0.1, and 2.0 M KCl, respectively (pH 7.4).*

In summary, the present study examined how a number of structurally diverse plant alkaloids (protoalkaloids (derivatives of β-phenylethylamine, benzylamines and colchicines), heterocyclic alkaloids (derivatives of purine, quinolysdine, piperidine, pyridine, quinoline and isoquinoline), and steroid alkaloids) alter the physical characteristics of lipid bilayers (membrane boundary potential and lipid packing) and properties of reconstituted ion channels formed by antimicrobial agents. We have provided clear evidence for alkaloid-mediated regulation of ion channel function by disturbing the lipid host. In addition to the possibility of the influence of alkaloids on nystatin channels by changing the elastic properties of the lipid matrix, especially by softening lipid bilayer, we have demonstrated for the first time the possibility of alkaloid-mediated regulation of peptide function by altering the distribution of the electric potential in the membrane. Specifically, we have shown the considerable effect of capsaicin and its close analog dihydrocapsaicin on the dipole potential of the membrane that plays a decisive role in the regulation of gramicidin A, syringomycin E and cecropin A channels. The dipole-modifying properties of piperine, theophylline, and synephrine, and the lipid disordering effects of piperine, tabersonine, and connesine should also be taken into the consideration of the possible mechanisms of their biological action.

## Materials and Methods

### Chemicals

All chemicals used were of reagent grade. Synthetic 1-palmitoyl-2-oleoyl-*sn*-glycero-3-phosphocholine (POPC), 1,2-dioleyl- *sn*-glycero-3-phosphoethanolamine (DOPE), 1,2-dioleyl-*sn*-glycero-3-phosphoserine (DOPS), 1,2-dipalmitoyl-*sn*-glycero-3-phosphocholine (DPPC), and cholesterol (Chol) were obtained from Avanti Polar Lipids (Pelham, United States).

Atropine (endo-(±)-α-(hydroxymethyl)benzeneacetic acid 8-methyl-8-azabicyclo[3.2.1]oct-3-yl ester), piperine ((*E,E*) -5- (3,4- methylenedioxyphenyl)-2,4-pentadienoylpiperidide), (-)-lupinine ([(1R,9aR)-2,3,4,6,7,8,9,9a-octahydro-1H-quinolizin -1-yl]methanol), (-)-cotinine ((*S*)-1-methyl-5-(3-pyridyl)-2- pyrrolidinone), berberine chloride (5,6-dihydro-9, 10- dimethoxybenzo[g]-1,3-benzodioxolo[5,6-a]quinolizinium), quinine (6′-methoxycinchonidine), melatonin (*N*-acetyl-5-methoxytryptamine), caffeine (1,3,7-trimethylxanthine), 1,7- dimethylxanthine, 3,9-dimethylxanthine, theophylline (1,3- dimethylxanthine), 3-isobutyl-1-methylxanthine, 7-(β- hydroxyethyl) theophylline (1,3-dimethyl-7-(2-hydroxyethyl) xanthine), pentoxifylline (3,7-dimethyl-1-(5-oxohexyl)xanthine), hordenine (4-(2-dimethylaminoethyl)phenol), (±)-synephrine (1-(4-hydroxyphenyl)-2-methylaminoethanol), colchicine ((*S*)- *N*-(5,6,7,9-tetrahydro-1,2,3,10-tetramethoxy-9-oxobenzo [a] heptalen-7-yl)acetamide), capsaicin (8-methyl-*N*-vanillyl- trans- 6-nonenamide), dihydrocapsaicin (8-methyl-*N*-vanillylno -nanamide), tabersonine ((5α,12β,19α)-2,3,6,7-tetrahydro-aspidospermidine-3-carboxylic acid methyl ester), solanidine ((3β)-solanid-5-en-3-ol), conessine ((3β)-*N*,*N*-dimethyl-con-5-enin-3-amine), nonactin, calcein, Triton X-100, Sephadex G-50, KCl, HEPES, DMSO, di-8-ANEPPS, gramicidin A (GrA), nystatin (Nys), and cecropin A (CecA) were purchased from Sigma Chemical (St. Louis, United States). The chemical structures of the alkaloids are presented in Figure 1.

KCl solutions (0.1, 1.0 or 2.0 M) was buffered using 5 mM HEPES, pH 7.4. Syringomycin E (SyrE) was isolated and purified as described previously (Bidwai and Takemoto, 1987) and was kindly offered by Dr. J.Y. Takemoto (Utah State University, United States). All experiments were performed at room temperature (25°C).

### Measurement of the Membrane Boundary and Dipole Potential

Changes in the steady-state conductance of planar lipid bilayers induced by complex of ionophore nonactin with potassium ion were measured to estimate the changes in the membrane boundary potential (φ_b_). Virtually solvent-free planar lipid bilayers were prepared using a monolayer-opposition technique (Montal and Muller, 1972) on a 50-μm-diameter aperture in a 10-μm-thick Teflon film separating two (*cis* and *trans*) compartments of a Teflon chamber. The aperture was pretreated with hexadecane. Lipid bilayers were made from POPC or equimolar mixture of DOPE and DOPS (DOPE/DOPS). After the membrane was completely formed and stabilized and its stability was assessed by applying voltages in the range from −200 to 200 mV with 50 mV-step for 5–10 min, stock solutions of nonactin A (7 μg/ml in ethanol) were added to the bathing solution (0.1 M KCl, 5 mM HEPES, pH 7.4) in both compartments to obtain a final concentration ranging from 0.1 to 1 μM. Ag/AgCl electrodes with 1.5% agarose/2 M KCl bridges were used to apply *V* and measure the transmembrane current *I*. Current was measured using an Axopatch 200B amplifier (Molecular Devices, LLC, Orlean, CA, United States) in the voltage clamp mode. Data were digitized using a Digidata 1440A and analyzed using pClamp 10.0 (Molecular Devices, LLC, Orlean, CA, United States). The conductance of the lipid bilayer was determined by measuring *I* at a constant transmembrane voltage (*V* = 50 mV). The K^+^-nonactin steady-state conductance was modulated via the two-sided addition of alkaloids from different mM stock solutions in water or ethanol to the membrane-bathing solution to obtain a final concentration ranging from 5 μM to 1 mM. The final concentration of ethanol in the chamber did not exceed 1% and the solvent does not affect membrane integrity.

The subsequent calculations were performed assuming that the membrane conductance is related to the φ_b_, the potential drop between the aqueous solution and the membrane hydrophobic core, by the Boltzmann distribution (Andersen et al., 1976).

(1) $$\Delta \,\varphi_{\text{b}}=\frac{k\,T}{e}\,\ln\left(\frac{G_{\text{m}}}{G_{\text{m}}^{0}}\right),$$

where *G*_m_ and *G_m_^0^* are the steady-state membrane conductances induced by K^+^-nonactin in the presence and absence of alkaloids, respectively; *e*, *k*, and *T* have their usual meanings.

The changes in φ_b_–value for the defined experimental conditions were averaged from 3 to 11 bilayers (mean ± sd).

A Langmuir adsorption isotherm was used to describe the adsorption of alkaloids to lipid bilayers in a first-order approximation as follows (de Levie et al., 1979; Reyes et al., 1983; Cseh et al., 2000; Efimova and Ostroumova, 2012; Ostroumova et al., 2013):

(2) $$\Delta \,\varphi_{\text{b}}\,\left(C\right)=\frac{\Delta \,\varphi_{\text{b}}\,\left(\infty \right)\,C}{C+K},$$

where Δφ_b_(C) is the boundary potential change at the *C* concentration of modifiers, Δφ_b_(∞) is the maximum potential change, and *K* is the desorption constant, which provides a meaningful measure of the affinity between the modifier and the lipid. The desorption constant can be determined as the slope of a linear dependence of [Δφ_b_(∞)]/[Δφ_b_(C)] on 1/*C*. The linear approximation of the obtained dependences were made using Origin 8.0 (OriginLab Corporation, Northampton, MA, United States).

The electric field distribution at the boundaries of the membrane consists of diffuse part of the electrical double layer (related to the presence of the charges on the membrane surface) and the potential drop over polar area inside the membrane itself (related to the dipolar components of lipids, interface water and other adsorbed molecules, and called membrane dipole potential, φ_*d*_).

To determine which component of the membrane boundary potential is responsible for the regulation of ion transport through membranes after the adsorption of alkaloids, the surface potential, or the dipole potential, φ_*d*_, the alteration in the φ_*d*_ of POPC membranes was estimated using a dipole-potential-sensitive lipid fluorescence probe, di-8-ANEPPS (Gross et al., 1994). Large unilamellar vesicles containing 1 mol% of di-8-ANEPPS were prepared by extrusion using an mini-extruder (Avanti Polar Lipids, Pelham, United States). POPC stock solution in chloroform was mixed with 50 μl of 1 mM di-8-ANEPPS in ethanol solution, twice resuspended in pentane and dried under a gentle stream of nitrogen. The dry lipid film was hydrated by buffer solution (0.1 M KCl, 5 mM HEPES-KOH, 1 mM EDTA, pH 7.4). The suspension was subjected to five freeze-thaw cycles and then passed by extrusion through a 100 nm nucleopore polycarbonate membrane 13 times to obtain unilamellar vesicles. Alkaloids were added to the liposome suspension to obtain a final concentration in the range from 200 μM to 1 mM. Steady-state fluorescence measurements were recorded with a Fluorat-02-Panorama spectrofluorometer (Lumex, Russia) at room temperature.

To avoid any effects of membrane fluidity alteration, the fluorescence excitation ratio *R* was defined as a ratio of the fluorescence intensity at an excitation wavelength of 420 nm to 520 nm at emission wavelength of 670 nm (Clarke and Kane, 1997). The changes in the φ_*d*_ of POPC bilayers were estimated using obtained values of *R* as described in Starke-Peterkovic et al. (2006). The changes in the φ_*d*_ for the defined experimental conditions were averaged from 3 to 7 bilayers (mean ± sd).

### Differential Scanning Calorimetry

Differential scanning calorimetry experiments were performed by a μDSC 7EVO microcalorimeter (Setaram, France). Giant unilamellar vesicles were prepared from pure DPPC by the electroformation method (standard protocol, 3 V, 10 Hz, 1 h, 55°C). The resulting liposome suspension contained 5 mM lipid and was buffered by 5 mM HEPES-KOH at pH 7.4. Alkaloids were added to aliquots to obtain a lipid:agent molar ratio of 10:1. The liposomal suspension was heated at a constant rate of 0.2 C/min. The reversibility of the thermal transitions was assessed by reheating the sample immediately after the cooling step from the previous scan. The temperature dependence of the excess heat capacity was analyzed using Calisto Processing (Setaram, France). The thermal behavior of the liposomes suspension in the absence and presence of the alkaloids was described by the changes in the temperature of the maximum of main phase transition (Δ*T*_m_) and width at a half height (half-width) of peak in the endotherm (Δ*T*_1/2_).

### Calcein Release From Large Unilamellar Vesicles

The alteration in the fluorescence intensity of calcein was used to monitor the membrane disintegration induced by alkaloids. Large unilamellar vesicles were prepared from pure POPC and mixture of POPC/Chol (67/33 mol%) by extrusion. The lipid stock in chloroform was dried under a gentle stream of nitrogen. The dry lipid film was hydrated using a buffer (35 mM calcein, 10 mM HEPES-NaOH, pH 7.4). The suspension was subjected to five freeze-thaw cycles and then passed through a 100 nm Nuclepore polycarbonate membrane 13 times. The calcein that was not entrapped in the vesicles was removed by gel filtration with a Sephadex G-50 column to replace the buffer outside the liposomes with calcein-free solution (150 mM NaCl, 1 mM EDTA, 10 mM HEPES-NaOH, pH 7.4). Calcein in vesicles fluoresces very poorly, because of strong self-quenching at millimolar concentrations, while the fluorescence of disengaged calcein in the surrounding media correlates to membrane stability and integrity in the absence and presence of alkaloids.

The time-dependence of calcein fluorescence de-quenching induced by 0.4 mM alkaloids had been measured for 80 min. The degree of calcein release was determined using a Fluorat-02-Panorama spectrofluorimeter (Lumex, Saint-Petersburg, Russia). The excitation wavelength was 490 nm and the emission wavelength was 520 nm. Addition of Triton X-100 from 10 mM water solution to a final concentration of 0.1 M to each sample led to complete disruption of vesicles and the intensity of fluorescence after releasing total amount of calcein from liposomes was measured.

To describe the dependence of permeabilization of the liposomal membranes on the concentrations of alkaloids, the relative intensity of calcein fluorescence (*RF*,%) was used:

(3) $$R\,F=\frac{I-I_{0}}{I_{\max}/0.9-I_{0}}\times 100\%,$$

where *I* and *I*_0_ are the calcein fluorescence intensity in the sample in the presence and absence of alkaloid, respectively, and I_max_ is the maximal fluorescence of the sample after lysis of liposomes by Triton X-100. A factor of 0.9 was introduced to account for the dilution of the sample by Triton X-100.

### Correlation Analysis

A correlation analysis was performed using Excel. Pearson’s correlation coefficient was applied to estimate the relationship between lipophilicity (Log*D*_o/w_ or Log*P*_o/w_) or polarity (μ) of alkaloid molecules and changes in the physical properties of lipid bilayers due to alkaloid adsorption (−Δφ_b_(max), −Δφ_*d*_(max), −Δ*T*_m_, Δ*T*_1/2_, *RF*_max_). To determine the confidence interval of the sample correlation coefficient, the Fisher transformation was used (a stated confidence level of 0.1, the sample size varied from 7 to 22 pairs, the minimum was determined by the available published data, and the maximum was determined by the number of tested compounds). The correlation coefficient was considered statistically significant if its confidence interval did not include 0. The Spearman’s rank correlation coefficients were also determined to confirm the significant correlation. Taking into account the linear relationship between the changes in the electric potential and the molecular dipole moment according to the equation for a parallel-plate capacitor, the Spearman criterion was not applied to μ and −Δφ_b_(max)/−Δφ_*d*_(max) relationships.

### Reconstitution of Ion Channels Into Planar Lipid Bilayers

Planar lipid bilayers were made by Montal and Muller (1972) from POPC, DOPE/DOPS or mixture of 67 mol% POPC and 33 mol% Chol. Pore-forming agents SyrE, CecA and Nys (from stock solution in water (pH 3.0, pH 7.4) and DMSO, respectively) were added to the aqueous phase on the *cis*-side of the bilayer to obtain a final concentration ranging from 0.5 to 1.0 μM of SyrE, 1 to 4 μM of CecA, and 7 to 20 μM of Nys. GrA was added to both compartments of the chamber up to 0.1–0.3 μM. The membranes modified by GrA, SyrE, CecA, and Nys were bathed in 2.0, 1.0, 0.1, and 2.0 M KCl, respectively (5 mM HEPES-KOH, pH 7.4). Alkaloids were added to both sides of the membrane up to 0.4 mM. The final concentration of DMSO in the chamber did not exceed 0.5% and did not produce any changes in the stability and ion permeability of the bilayers. The tested alkaloids in the concentrations used did not increase the ion permeability of lipid bilayers in the absence of ionophore and pore-forming agents.

«Positive» voltage refers to the case in which the *cis*-side compartment is positive with respect to the *trans*-side. Data were acquired at a sampling frequency of 5 kHz using low-pass filtering at 200 Hz, and the current tracks were processed through an 8-pole Bessel 100-kHz filter. Single-channel conductance (*G*_SC_) of GrA and SyrE channels was defined as the ratio between the current flowing through a single channel (*i*) and *V*. The dwell time of the single channels (τ) to be in an open state was determined using pClamp 10. The total numbers of events used for the channel conductance fluctuation and dwell time analysis were 1000–1500 and 1500–2000, respectively. Peaks on the conductance and dwell time histograms were fitted by the normal density and exponential functions, respectively. The χ^2^ criterion was applied (*P* < 0.05). The mean ratio (*I*_∞_/*I_∞_^0^*) of the macroscopic currents after (*I*_∞_) and before (*I_∞_^0^*) two-sided modifier addition of tested alkaloids was used to assess the changes in channel-forming activity of CecA and Nys.

The characteristic parameters of channel-forming activity of GrA, SyrE, CecA, and Nys in the presence of tested modifiers were averaged from 5 to 9 bilayers (mean ± sd). All experiments were performed at room temperature.

## Data Availability Statement

The original contributions presented in the study are included in the article/Supplementary Material, further inquiries can be directed to the corresponding author/s.

## Author Contributions

OO contributed to the conceptualization, project administration, funding acquisition, and reviewing and editing the manuscript. SE contributed to the investigation, analysis, validation, and writing the original draft of manuscript. AZ contributed to the investigation and analysis. All authors contributed to the article and approved the submitted version.

## Conflict of Interest

The authors declare that the research was conducted in the absence of any commercial or financial relationships that could be construed as a potential conflict of interest.
