# Supplementary material for: Alkaloids Modulate the Functioning of Ion Channels Produced by Antimicrobial Agents via an Influence on the Lipid Host
Source: Front Cell Dev Biol. 2020 Jun 30;8:537. doi: 10.3389/fcell.2020.00537 (PMC7339123; doi:10.3389/fcell.2020.00537)
Supplement: Supplementary file 1 [file Data_Sheet_1.pdf]

Supplementary Material

1.1 Supplementary Figures

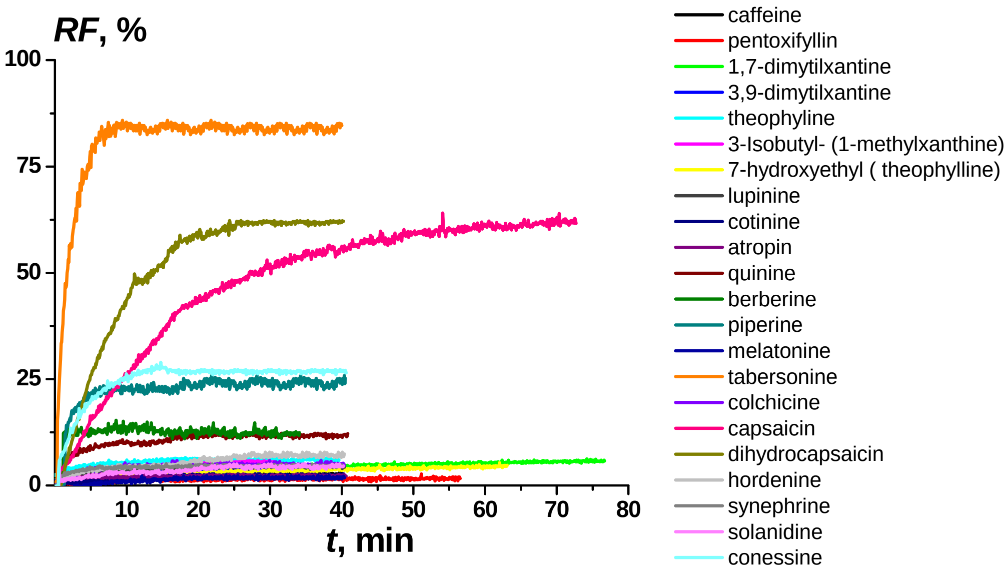

Supplementary Figure 1. The time dependence of relative fluorescence of calcein (*RF*,%) leaked from POPC vesicles. Alkaloids were added into the liposomal suspension up to a concentration of 0.4 mM at the first time point. The relationship between the color line and the compound is given in the figure.

1.2 Supplementary Tables

Supplementary TABLE 1S. The physicochemical properties of tested alkaloids: *pKa* – the logarithm of acid dissociation constant, $LogD_{o/w}$ – the logarithm of octanol/water distribution coefficient at pH 7.4, $LogP_{o/w}$ – the logarithm of octanol/water partition coefficient, μ – molecule dipole moment.

Alkaloid	*pKa**		$LogD_{o/w}$*	$LogP_{o/w}$[#]	Reference	$\mu^{\$}$, D	$\mu^{\#}$, D	Reference
	acidic	*basic*						
caffeine		-0.92	-0.55	-0.07, -0.08	Gaspari and Bonati, 1987; Lombardo et al., 2001; Kupsakova et al., 2004; Machatha and Yalkowsky, 2005; Schonsee and Bucheli, 2020	3.29	4.58, 4.23	Aaron and Gaye, 1987; Fischer et al., 1999
pentoxifylline	19.64	-0.93	0.23	0.29, 0.12	Lombardo et al., 2001; Hollosy et al., 2006	5.44	4.78	Augusto and Quintanilla, 2010
1,7-dimethylxanthine	10.76	-0.87	0.24	-0.24	Gaspari and Bonati, 1987	3.59	*na*	–
3,9-dimethylxanthine	8.99	1.09	-0.82	*na*		7.29	*na*	–
theophylline	7.82	-0.78	-0.89	-0.04, -0.02	Gaspari and Bonati, 1987; Kupsakova et al., 2004; Machatha and Yalkowsky, 2005	6.53	4.64, 7.66	Aaron and Gaye, 1987
3-isobutyl-1-methylxanthine	11.41	2.42	0.40	1.46[&]	Kupsakova et al., 2004	6.99	*na*	–
7-(β-hydroxyethyl) theophylline	15.43	-1.25	-1.24	*na*	–	2.26	*na*	–
lupinine	15.42	9.88	-1.52	*na*	–	1.24	*na*	–
cotinine		4.79	0.21	0.07	Li et al., 1992	4.95	*na*	–
atropine	15.15	9.39	-0.41	-0.55, 1.32, 1.82	Lombardo et al., 2001; Kuo et al., 2004; Machatha and Yalkowsky, 2005	3.58	4.39	Kuo et al., 2004

quinine	13.89	9.05	0.86	2.36, 2.79	Machatha and Yalkowsky, 2005; Hollosy et al., 2006	2.39	2.17	Karle and Bhattacharjee, 1999
berberine		*na*	-1.28	-1.03, -1.5	Schonsee and Bucheli, 2020; Wang et al., 2012	*na*	*na*	–
piperine		-0.13	2.78	2.25	Wu et al., 2012	5.37	5.98, 6.36	Gokalp, 2016
melatonin	15.8	-1.60	1.15	1.39, 1.2	Yu et al., 2016; Kanikkannan et al., 2001	4.93	4.76, 6.29	Zavodnik et al., 2006; Galijasevic and Hodzic, 2012
tabersonine	14.07	9.08	0.90	*na*	–	1.28	*na*	–
colchicine	15.06	-0.04	1.46	1.14, 1.2	Schonsee and Bucheli, 2020; Hollosy et al., 2006	6.53	*na*	–
capsaicin	9.93	-0.52	3.75	3.8	Hanson et al., 2015	4.66	3.96	Pereira et al., 2019
dihydrocapsaicin	9.93	-0.52	4.11	*na*	–	4.95	*na*	–
hordenine	10.31	9.19	0.06	*na*	–	0.99	1.49	Dwivedi et al., 2014
synephrine	9.76	9.15	-1.39	-0.45	Hansch and Zhang, 1995	2.32	2.82[@]	Cabezas et al., 2013
solanidine	18.2	12.47	1.39	*na*	–	1.24	*na*	–
conessine		10.53	-1.45	*na*	–	1.81	*na*	–

*The values of pKa and $LogD_{o/w}$ were predicted by ChemAxon

[$]Calculations of the dipole moments of the alkaloid molecules were performed by HyperChem 7.0 (Hypercube, Inc., Gainesville, FL, USA) using the semi-empirical MNDO

[#]Available literature data

[&]Partition coefficient of 1-isobutyl-3-methylxanthine

[@]Mean dipole moment of low-energy conformers

na = Not available

1.3 Supplementary References

Aaron, J.J., Gaye, M.D. (1987). Experimental and theoretical dipole moment of purines in their ground and lowest excited singlet states. J. Mol. Struct. 156, 119-135.

Augusto, V., Quintanilla, Y. (2010). Rejection of emerging organic contaminants by nanofiltration and reverse osmosis membranes: effects of fouling, modelling and water reuse. 1st Edition, P.216, doi:10.1201/b10832.

Cabezas, C., Simão, A., Bermúdez, C., Varela, M., Peña, I., Mata, S., Fausto, R., Alonso, J.L. (2013). Conformational analysis of octopamine and synephrine in the gas phase. J. Phys. Chem. A. 117, 4907-4915. doi:10.1021/jp4032223.

Dwivedi, A., Dubey, V., Bajpai, A.K. (2014). A quantum chemical comparative study of epinine and hordenine. Int. J. Chem. Stud. 2, 20-26.

Fischer, B., Yefidoff, R., Major, D.T., Rutman-Halili, I., Shneyvays, V., Zinman, T., Jacobson, K.A., Shainberg, A. (1999). Characterization of "mini-nucleotides" as P2X receptor agonists in rat cardiomyocyte cultures. An integrated synthetic, biochemical, and theoretical study. J. Med. Chem. 42, 2685-2696. doi:10.1021/jm990085i.

Galijasevic, S., Hodzic, E. (2012). Theoretical studies of structures and thermodynamic parameters of melatonin and its metabolites: N1-Acetyl-N2-formyl-5-metoxy kynuramine and N1-Acetyl-5-metoxykynuramine. Bull. Chem. Technol. Bosn. Herzeg. 39, 39-43.

Gaspari, F., Bonati, M. (1987). Correlation between n-octanol/water partition coefficient and liquid chromatographic retention for caffeine aid its metaboliyes: and some structure-pharmacokinetic considerations. J. Pharm. Pharmacol. 39, 252-260.

Gökalp, F. (2016). A study on piperine, active compound of black pepper. Acad. Plat. J. Engineer. Sci. 4, 18-24. doi:10.21541/apjes.66230

Hansch, C., Zhang, L. (1995). Comparative QSAR: radical toxicity and scavenging. Two different sides of the same coin. SAR QSAR Environ. Res. 4, 73-82. doi:10.1080/10629369508029905.

Hanson, S.M., Newstead S., Swartz, K.J., Sansom, M.S.P. (2015). Capsaicin interaction with TRPV1 channels in a lipid bilayer: molecular dynamics simulation. Biophys. J. 108, 1425-1434. doi:10.1016/j.bpj.2015.02.013.

Hollosy, F., Valko, K., Hersey, A., Nunhuck, S., Keri, G., Bevan, C. (2006). Estimation of volume of distribution in humans from high throughput HPLC-based measurements of human serum albumin (HSA) binding and immobilized artificial membrane (IAM) partitioning. J. Med. Chem. 49, 6958-6971. doi:10.1021/jm050957i.

Kanikkannan, N., Jackson, T., Shaik, M.S., Singh, M. (2001). Evaluation of skin sensitization potential of melatonin and nimesulide by murine local lymph node assay. Eur. J. Pharm. Sci. 14, 217-220. doi:10.1016/s0928-0987(01)00176-2.

Karle, J.M., Bhattacharjee, A.K. (1999). Stereoelectronic features of the cinchona alkaloids determine their differential antimalarial activity. Bioorg. Med. Chem. 7, 1769-1774. doi:10.1016/s0968-0896(99)00120-0.

Kuo, C.L., Wang, R.B., Shen, L.J., Lien, L.L., Lien, E.J. (2004). G-protein coupled receptors: SAR analyses of neurotransmitters and antagonists. J. Clin. Pharm. Ther. 29, 279-298. doi:10.1111/j.1365-2710.2004.00563.x.

Kupsakova, I., Rybár, A., Docolomanský, P., Drobná, Z., Stein, U., Walther, W., Barancík, M., Breier, A. (2004). Reversal of P-glycoprotein mediated vincristine resistance of L1210/VCR cells by analogues of pentoxifylline A QSAR study. Eur. J. Pharm. Sci. 21, 283-293. doi:10.1016/j.ejps.2003.10.019.

Li, N.Y., Li, N., Gorrod, J.W. (1992). Determination of partition coefficients and ionisation constants of (S)(-)-nicotine and certain metabolites. Med. Sci. Res. 20, 901-902.

Lombardo, F., Shalaeva, M.Y., Tupper, K.A., Gao, F. (2001). ElogDoct: a tool for lipophilicity determination in drug discovery. 2. Basic and neutral compounds. J. Med. Chem. 44, 2490-2497. doi:10.1021/jm0100990.

Machatha, S.G., Yalkowsky, S.H. (2005). Comparison of the octanol/water partition coefficients calculated by ClogP®, ACDlogP and KowWin® to experimentally determined values. Int. J. Pharm. 294, 185-192. doi:10.1016/j.ijpharm.2005.01.023.

Pereira, G.J.V., Tavares, M.T., Azevedo, R.A., Martins, B.B., Cunha, M.R., Bhardwaj, R., Cury. Y,, Zambelli, V.O., Barbosa, E.G., Hediger, M.A., Parise-Filho, R. (2019). Capsaicin-like analogue induced selective apoptosis in A2058 melanoma cells: Design, synthesis and molecular modeling. Bioorg. Med. Chem. 27, 2893-2904. doi:10.1016/j.bmc.2019.05.020.

Schonsee, C.D., Bucheli, T.D. (2020). Experimental determination of octanol−water partition coefficients of selected natural toxins. J. Chem. Eng. Data. 65, 1946−1953. doi:10.1021/acs.jced.9b01129

Wang, J.R., Tanaka, T., Zhang, H., Kouno, I., Jiang, Z.H. (2012). Formation and conformation of baicalin–berberine and wogonoside–berberine complexes. Chem. Pharm. Bull. (Tokyo). 60, 706-711. doi:10.1248/cpb.60.706.

Wu, Z.J., Xia, X.J., Huang, X.S. (2012). Determination of equilibrium solubility and apparent oil/water partition coefficient of piperine. J. Jinan Univ.(Nat. Sci. & Med. Edit.). 2012-05.

Yu, H., Dickson, E.J., Jung, S.R., Koh, D.S., Hille, B. (2016). High membrane permeability for melatonin. J. Gen. Physiol. 147, 63–76. doi:10.1085/jgp.201511526.

Zavodnik, I.B., Domanski, A.V., Lapshina, E.A., Bryszewska, M., Reiter, R.J. (2006). Melatonin directly scavenges free radicals generated in red blood cells and a cell-free system: chemiluminescence measurements and theoretical calculations. Life Sci. 79, 391-400. doi:10.1016/j.lfs.2006.01.030.
